# Paper-based microfluidic devices for electrochemical quantification of the explosive picric acid

**DOI:** 10.1007/s00604-026-08311-0

**Published:** 2026-07-31

**Authors:** Julia de Oliveira Cardoso, Lauro Antonio Pradela Filho, Thiago Regis Longo Cesar da Paixão

**Affiliations:** 1 Department of Fundamental Chemistry, Institute of Chemistry, University of São Paulo, São Paulo, SP 05508-000 Brazil; 2 Institute of Chemistry, Federal University of Mato Grosso Do Sul, Campo Grande, MS 79070-900 Brazil

**Keywords:** Explosive, Microfluidic, Paper-based device, 3D printing, Forensic, Environmental

## Abstract

A high-throughput analytical method for quantifying the explosive picric acid (PA) is reported. The approach first involves developing a three-electrode thermoplastic chip, which was then integrated with circular paper structures to generate microfluidic paper-based analytical devices (µPADs). The electrochemical setup was fabricated using a 3D printing pen, a tool commercialized as a toy for kids. The electrochemical chips were initially tested using differential pulse voltammetry to promote the analyte's reduction. After evaluating the PA electrochemical behavior, the µPADs were assembled using a practical procedure with clamps and filter paper. The µPAD assembly was optimized by evaluating the paper substrate, injection volume, and detection potential. Larger-pore papers resulted in a superior response due to greater accessibility of the analyte solution to the electrode surface during injection. Increasing the injection volume enhanced the signal intensity, besides providing better repeatability. Increasing the detection potential enhances the system response. However, this parameter affects selectivity as other species could also be reduced on the electrode surface. Under optimal conditions, the proposed system enabled the sequential injection of an analyte solution into a single device, yielding a sample throughput of (131 ± 14) injections h^−1^. Furthermore, a linear response was obtained from 10 to 100 µmol L⁻^1^ picric acid, with a limit of detection of 5.0 µmol L^−1^. Moreover, the system's applicability was further demonstrated for lake water analyses through recovery studies. Therefore, this work reports a practical analytical tool for the PA quantification in environmental analyses.

**Graphical Abstract:**

**Figure:**
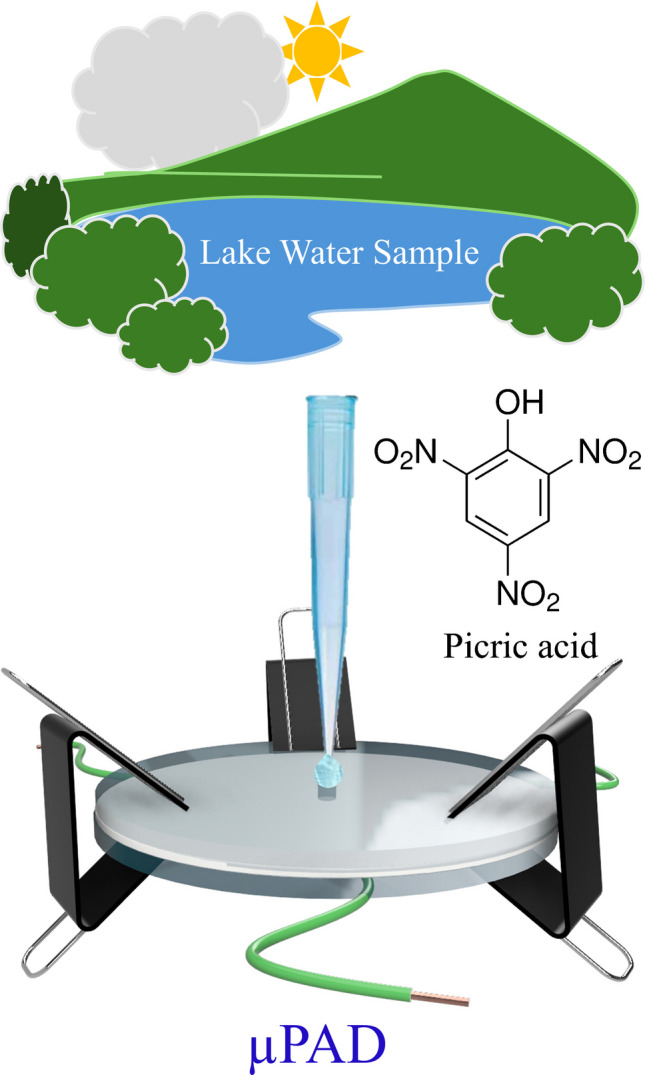

**Supplementary Information:**

The online version contains supplementary material available at 10.1007/s00604-026-08311-0.

## Introduction

Nitroaromatic compounds are widely used in industry, serving as fundamental components of dyes, agrochemicals, plastics, explosives, and pharmaceuticals [1, 2]. Among them, 2,4,6-trinitrophenol, known as picric acid (PA), is highly reactive and flammable. This compound has been historically used in the production of weapons and explosives [3]. In general, explosive compounds have been used in mining and military activities. Additionally, they have been illegally used by criminal organizations and terrorist groups to conduct violent acts and destabilize society. Despite serving a military purpose, some compounds are highly toxic and carcinogenic, [1] making them environmental pollutants. PA is also a highly water-soluble compound, causing serious implications to human health, such as liver malfunction, skin and eye irritation, and chronic diseases, such as cancer, anemia, and cyanosis [4]. Therefore, developing analytical tools for PA quantification has become a highly attractive approach, considering the importance of public health and environmental safety. Such analytical tools could also assist investigative police in analyzing suspect materials in decentralized areas, where access to conventional laboratory infrastructure is limited.

Various techniques have been used for PA quantification, including chromatography [5], molecular absorption spectrophotometry [6], and fluorescence [7, 8]. However, most of these methods rely on complex derivatization steps and expensive instrumentation, restricting analysis at the point of need. In this sense, electrochemical devices have emerged as promising tools for on-site detection, as they provide rapid responses [9]. Additionally, electrochemical sensors can be inexpensively fabricated at a miniaturized size, enabling their integration with portable instrumentation for out-of-lab analysis [10].

The electrochemical quantification of picric acid has been studied on different electrode surfaces, including screen-printed electrodes [11], inkjet-printed electrodes [12, 13], and thermoplastic electrodes [14]. These electrodes differ mainly in the fabrication process. The screen printing fabrication consisted of using conductive ink and a plastic mask with an electrode layout [11]. This mask is placed over the electrode substrate, followed by spreading the conductive ink over this substrate with a squeegee. This mask is subsequently removed, and the ink dries, resulting in the disposable electrochemical sensors. Although screen printing produces cost-effective devices, this technique requires the adjustment of ink viscosity, which typically relies on organic solvents. However, prolonged exposure to these solvents during the electrode fabrication may cause health risks to the sensor maker. Furthermore, solvent evaporation during processing can alter the ink composition, leading to batch-to-batch variability and potentially compromising sensor performance [15].

Unlike screen printing, the main advantage of inkjet printing is its automated nature [12, 13], eliminating the manual dependence on the sensor maker during electrode fabrication. Despite the advantages, clogging of the cartridge is a common problem faced with this technique, especially when using high-viscosity or unstable inks [15]. To replace ink-based techniques, fused deposition modeling (FDM), a 3D printing technique, has been widely explored for electrode fabrication [16–20]. Such a technique relies on extruding a semi-molten thermoplastic filament through a heated nozzle. The extruded material is sequentially deposited in a layer-by-layer format, with the stacked layers resulting in the desired design [21]. Palenzuela et al. [14] developed 3D-printed electrodes for voltammetric determination of picric acid. The electrodes were fabricated with a graphene/polylactic acid filament using a desktop 3D printer. In that work, the authors activated the electrode surface by immersion in dimethylformamide (DMF) for 10 min. The activation step enhanced the electrode response for different redox probes, such as Fc-COOH, K_3_Fe(CN)_6_/K_4_Fe(CN)_6_, FeCl_3_, and ascorbic acid. The enhancement was associated with the dissolution of the fused polymer, exposing the graphene particle on the electrode surface. Besides subjecting the 3D-printed electrodes to chemical activation, other works have also explored the effect of electrochemical [22], thermal [23], plasma [24], and laser treatments [25], making these steps essential for electrode use.

Despite the achievements, fabricating electrodes with a desktop 3D printer requires well-trained personnel to design 3D objects and control printing conditions [26, 27]. To overcome this, 3D printing pens have been explored for this purpose [26, 28]. This tool simplifies the electrode processing, as the fabrication process relies exclusively on a mold during filament extrusion [17]. In this sense, Pradela-Filho et al. [27] recently developed thermoplastic electrodes based on polylactic acid (PLA) and carbon black (CB) filament using a 3D printing pen. The authors demonstrated that the resulting sensor dispenses the need for electrode activation, due to improvement of the electrochemical response by adjusting the thickness and diameters of the electrodes, generating solid structures with good electrical contacts. The resulting sensors were satisfactorily applied to the quantification of organic and inorganic species, including paraquat, Pb^2+^, and caffeic acid.

In this context, the present work will explore the utility of PLA/CB electrodes for picric acid quantification. One of the goals is to continue evaluating the applicability of these systems in the absence of (electro)chemical treatment, validating our recent findings [27] and expanding the sensors' applicability. Moreover, the idea is to evaluate the system's versatility for fluidic analysis. Junqueira et al. [3] quantified picric acid using a copper electrode through flow injection analysis (FIA) system. Compared to conventional systems, the FIA system reduces reagent and sample consumption while enhancing the sample throughput. However, this system relies on external pumps, increasing the system complexity and restricting miniaturization. To overcome this, our work also proposes the combination of a three-electrode electrochemical chip with paper structures, generating microfluidic paper-based analytical devices (µPADs).

The µPADs are inspired by the batch-injection analysis (BIA) system [29], as previously reported by Arantes et al. [30]. Unlike their work, our system is fabricated with thermoplastic filaments and a 3D printing pen. Additionally, the system is entirely enclosed with polymethyl methacrylate (PMMA), which helps prevent contamination during device and solution handling. Additionally, the sample solution is introduced in the center of the device, which might enhance the solution's spreading due to the radial flow. Therefore, this work introduces a practical alternative for µPAD fabrication, with a focus on picric acid quantification in lake water samples.

## Experimental

### Reagents and materials

All solutions were prepared using ultrapure water (Direct-Q 5 Ultrapure Water Systems, Millipore, Massachusetts). The pH study was performed using Britton-Robinson (BR) buffer solution, prepared by mixing glacial acetic acid (Vetec), boric acid (Vetec), and monosodium phosphate (Vetec), all at a concentration of 0.04 mol L⁻^1^. Hydrochloric acid (Synth) and potassium hydroxide (Nuclear) solutions were used to adjust the pH value of the buffer solutions. Picric acid was purchased from Reagen (Rio de Janeiro, Brazil) and nitrobenzene from Carlo Erba (Milano, Italy). Standard picric acid solutions were prepared daily for electrochemical studies. The copper wires, sandpapers, and epoxy glue were purchased from local markets.

### Fabrication of electrochemical chips

**Fig. 1:**
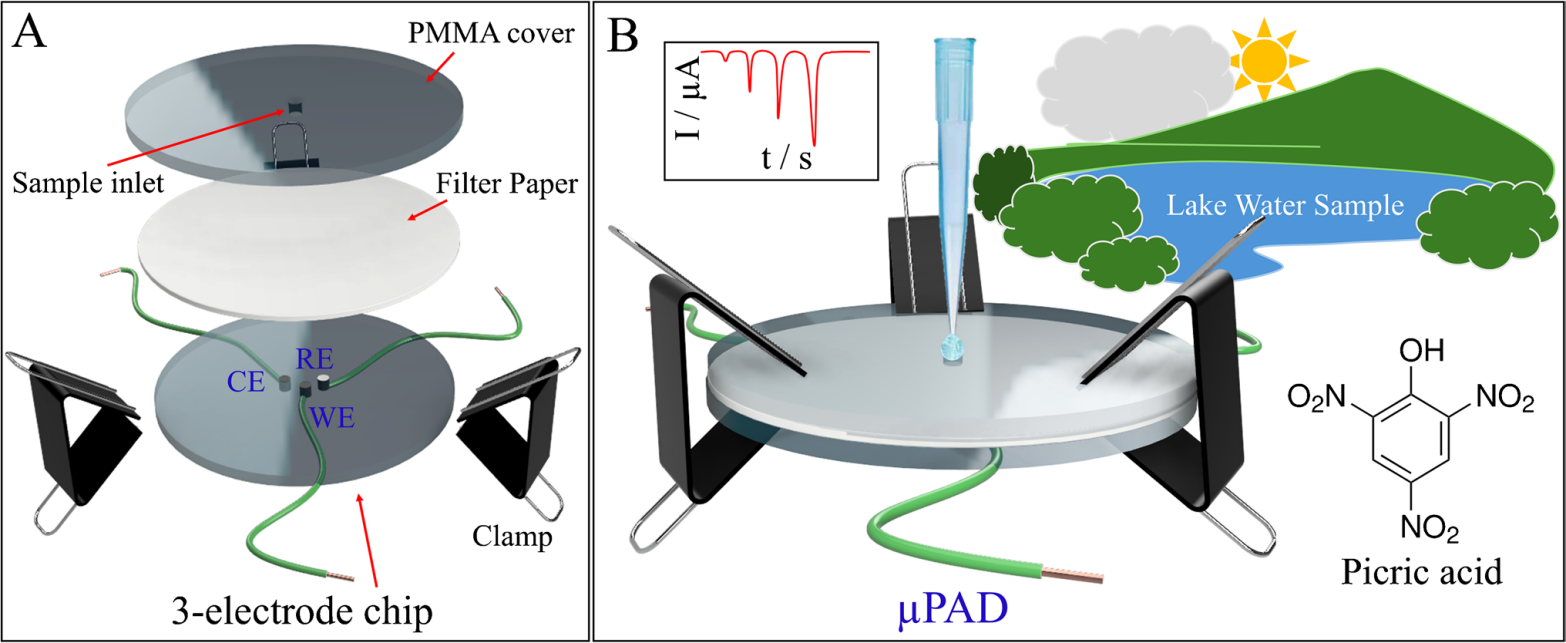
Assembling process and analytical application of the resulting µPADs. **A** µPAD components. **B** Scheme of the device's applicability for lake water analysis

The electrochemical chip (Fig. 1A) consists of three integrated electrodes, with each having a diameter of 2 mm. They are called the working electrode (WE), the counter electrode (CE), and the reference electrode (RE). The WE was fabricated at the center of this electrochemical chip, with RE and CE fabricated 3 mm away from the WE. This system was made on a PMMA plate (1 mm thickness). This plate was cut with a CO₂ laser (Work Special Laser) to produce the three-electrode mold. The system geometry was created in RDWorks 8.0. The 3D-printing pen, equipped with a 0.7 mm diameter nozzle, was used to fill the PMMA mold with the conductive filament (Protopasta). Waterproof sandpapers with 150 and 600 grit were used to remove excess filaments from the PMMA mold. Copper wires were then attached to the back of the PMMA mold using the conductive filament and epoxy glue. These wires were necessary for connecting the electrodes to the electrochemical instrumentation.

Preliminary studies (data not shown) were conducted with carbon black/polylactic acid filament RE. However, the voltammetric profile of picric acid had a shift in reduction potential when testing different electrochemical chips. To circumvent this issue, silver glue (Joint Metal Ltda) was applied to the reference electrode. Then, 10 µL of sodium hypochlorite (15%) was left on the painted RE for 2 min, generating an Ag/AgCl-pseudo RE [31, 32]. The resulting systems were initially characterized under static conditions and later coupled to paper-based microfluidic structures.

Electrochemical measurements were performed using an Autolab PGSTAT128N potentiostat (Eco Chemie, Utrecht, The Netherlands). This equipment was connected to a microcomputer running the NOVA 2.1.4 software.

### µPAD fabrication

Figure 1A also shows the µPAD assembly, highlighting its components. Chromatographic paper and Whatman 40 (W40), Whatman 41 (W41), and Whatman 42 (W42) quantitative filter papers were evaluated as substrates in the device assembly. These papers were cut into circles (50 mm in diameter) using a CO₂ laser machine. The circular paper was then attached to the three-electrode electrochemical system. A PMMA cover (50 mm in diameter) containing a central hole (2 mm in diameter) was placed over the circular paper previously positioned on the electrodes. The hole serves as a sample inlet (Fig. 1A). Finally, the system was closed using paper clamps with constant pressure. Electrochemical measurements were conducted using amperometry.

### Lake water analyses

The proposed µPADs were used to analyze lake water samples (Fig. 1B). The analyses were performed using 0.1 mol L^−1^ HCl as the supporting electrolyte, and µPADs fabricated with W41 filter paper. Amperometry was used as an analytical technique, applying a detection potential of −0.5 V *vs.* Ag/AgCl-pseudo RE. Sample injection was performed using a conventional micropipette, with an optimized injection volume of 2 µL.

The water sample was collected at the lake of the main campus of the University of São Paulo (São Paulo—SP). To avoid sample dilution, the HCl solution was prepared directly in the tap water sample instead of using ultrapure water, resulting in a final HCl concentration of 0.1 mol L^−1^ in the tap water sample. The system was calibrated using the external standard method. This calibration was performed with standard solutions of 10, 25, 50, 75, and 100 µmol L^−1^ picric acid. To obtain the blank solution, a supporting electrolyte solution (HCl 0.1 mol L^−1^) was prepared in ultrapure water. The analytical signals of the standard solutions were subtracted from the blank signal to plot the calibration curve. Although the system could also respond to higher analyte concentrations, the linearity was only evaluated up to 100 µmol L^−1^ since a simple dilution could enable the analysis of samples contaminated with higher analyte concentrations.

The analyses were performed by recovery studies, in which the tap water sample was spiked with picric acid at concentration levels of 50 and 100 µmol L^−1^. Given the absence of picric acid in the lake water, the analyses were conducted by first injecting the blank and standard solutions, followed by the injection of the spiked lake water samples. After sample injection, their respective current intensities were also subtracted from the blank, and the resulting values were used to calculate the picric acid concentration from the calibration curve equation.

## Results and discussions

### Voltammetric characterization

Despite the importance of nitroaromatic compounds for military and mining activities, they are restricted materials because of their high toxicity and illegal use by criminal organizations. In this context, three-electrode thermoplastic chips were fabricated for PA quantification, with environmental applications in mind. The thermoplastic chips were fabricated through a straightforward strategy. The fabrication consists basically of using a 3D printing pen to fill PMMA templates with commercial conductive filament, generating electrochemical chips. It is important to highlight that the 3D printing pen is commercialized as a children's toy, costing approximately 15 dollars on the Amazon website. Besides simplifying the electrode fabrication, the resulting chips are miniaturized, requiring only a few µL of supporting electrolyte solution for electrochemical measurements, which significantly reduces the waste compared to traditional analysis.

**Fig. 2:**
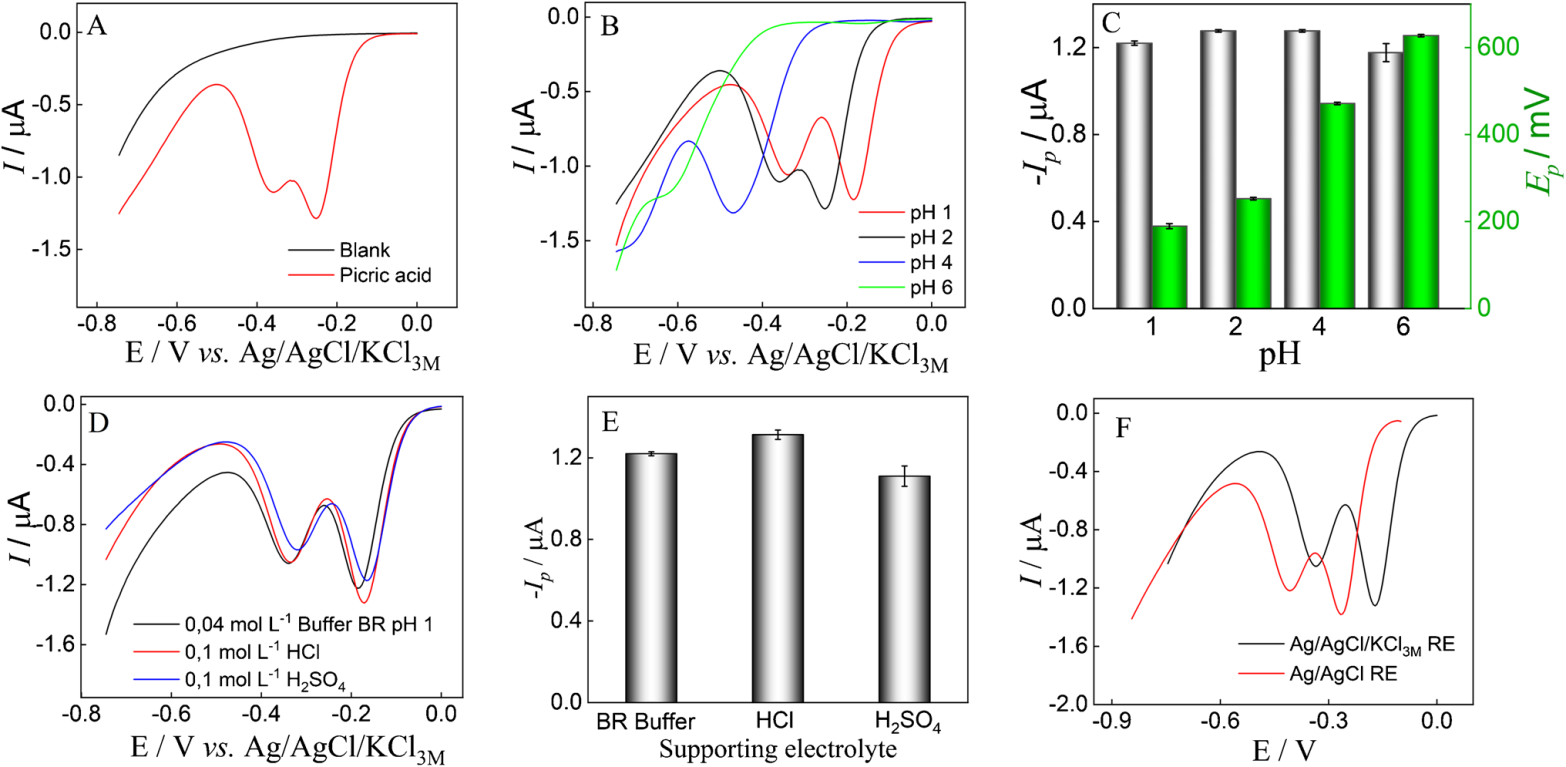
Evaluation of the sensor response for picric acid, followed by pH study, supporting electrolyte selection, and effect of the reference electrode type. **A** Differential pulse voltammograms (DPVs) were recorded with 0.04 mol L^−1^ buffer BR pH 2 in the absence and presence of 50 µmol L^−1^ picric acid. **B** DPVs recorded with 0.04 mol L^−1^ buffer BR in pH values from 1 to 6. Picric acid concentration: 50 µmol L^−1^. **C** Respective graph of *-I*_*p*_ and *E*_*p*_* vs.* pH value. **D** DPVs were recorded with different electrolyte solutions, such as HCl, H_2_SO_4_, and buffer BR. **E** Respective graph of *-I*_*p*_ vs supporting electrolyte type. **F** DPVs recorded with different reference electrodes, such as Ag/AgCl-pseudo RE and a conventional reference electrode (Ag/AgCl/KCl/3 M RE). Step: 5 mV. Amplitude: 75 mV. Modulation time: 0.1 s. Time step: 0.5 s

The resulting chips were initially characterized by differential pulse voltammetry using a conventional reference electrode (Ag/AgCl/KCl_3M_ RE), with results shown in Fig. 2A. PA is reduced on the thermoplastic electrode surface, providing a couple of voltammetric peaks. Additionally, this process occurs (Fig. 2A) within a potential range free from oxygen interference under acid conditions, which can be advantageous for amperometric measurements. One reduction mechanism proposed for three-nitro explosives involves the sequential conversion of the nitro groups into hydroxylamine (Eq. 1), followed by reduction to the amine groups (Eq. 2) [3, 12].

1 $$RN{O}_{2}+4{H}^{+}+4{e}^{-}\rightleftharpoons RNHOH+{H}_{2}O$$

2 $$RNHOH+2{H}^{+}+2{e}^{-}\rightleftharpoons RN{H}_{2}+{H}_{2}O$$

According to the literature [3], this first reaction (Eq. 1) can also occur via two steps under acidic conditions. The nitro group (Eq. 3) is reduced to a radical anion (RNO_2_^•−^), which is then reduced to a hydroxylamine involving three electrons and four protons (Eq. 4).

3 $$RN{O}_{2}+{e}^{-}\rightleftharpoons RN{O}_{2}^{\bullet -}$$

4 $$RN{O}_{2}^{\bullet -}+4{H}^{+}+3{e}^{-}\rightleftharpoons RNHOH+{H}_{2}O$$

Considering these reactions (Eqs. 1 - 4), the voltammetric peaks (Fig. 2A) of picric acid have been attributed to the stepwise reduction of the nitro groups through radical and hydroxylamine intermediates, yielding the corresponding amine derivatives [33]. Despite the proposed mechanisms, the reduction process of picric acid is more complex than these simplified reactions, as the literature also shows that this redox process can produce from one to four cathodic peaks depending on the electrode type and experimental conditions [3, 33, 34], suggesting that multiple reduction pathways may occur under different electrochemical conditions.

The voltammetric response of PA was next evaluated at different pH values (Fig. 2B). Figure 2C represents the peak current (*I*_*p*_) and peak potential (*E*_*p*_) obtained from the respective voltammograms (Fig. 2B). *I*_*P*_ was practically the same across pH values. However, *E*_*p*_ remarkably decreased upon varying the pH values from 6 to 1, demonstrating that the reduction process of the analyte is facilitated in an acidic medium. Junqueira et al. [3] observed the same behavior for PA reduction at copper electrode surfaces. They reported that increasing the pH values shifted the reduction potential towards more negative values. This was associated with the deprotonation of the PA structure (pK_a_ = 0.38) at higher pH values. Under this condition, the negatively charged structures can stabilize the nitro groups, making the reduction process more difficult. Considering this result, pH 1 was selected for subsequent studies.

The voltammetric response of PA was also evaluated in different supporting electrolyte solutions (Fig. 2D) at pH 1. The solutions include HCl, H_2_SO_4_, and buffer BR. Figure 2E shows that the supporting electrolyte has no substantial effect on the electrochemical response for PA. Consequently, HCl 0.1 mol L^−1^ was selected as the supporting electrolyte because of the simplicity of the preparation. After evaluating the supporting electrolyte, the electrochemical behavior of PA was assessed with the Ag/AgCl pseudo RE and conventional RE. Figure 2F shows that the PA reduction peak shifted toward more negative potentials (approximately −100 mV) with the Ag/AgCl pseudo-RE. Additionally, this electrode did not cause any distortion in the voltammetric profiles, suggesting Ag-pseudo RE can be utilized for electrochemical measurements. It is important to mention that the proposed system responded to PA without the need for any electrochemical or chemical activation step, which is a common prerequisite for 3D-printed electrodes. This result aligns with our previous findings [27], demonstrating that 3D pen-extruded electrodes dispense any additional (electro)chemical treatments for analytical applications.

### µPAD optimization

**Fig. 3:**
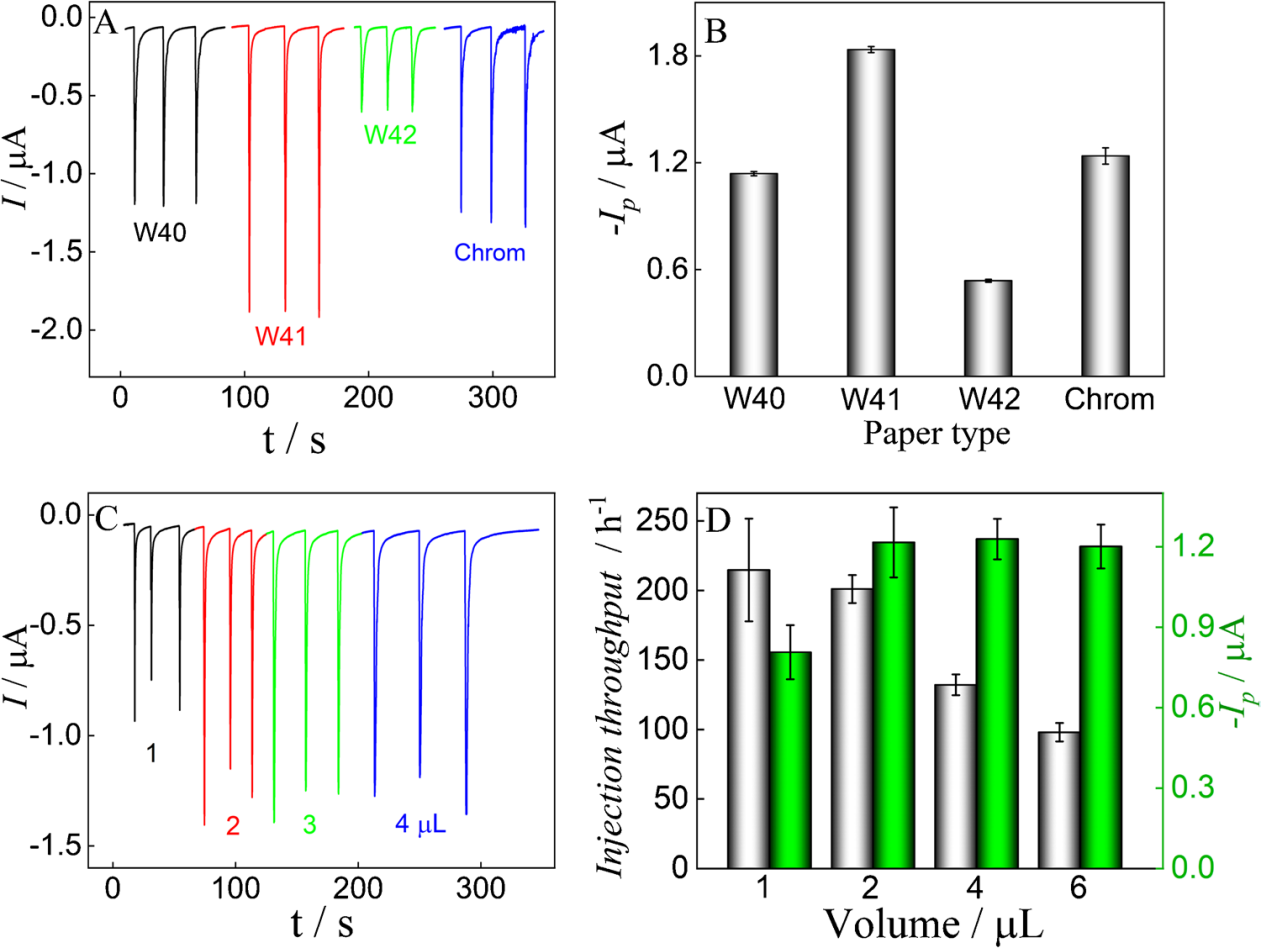
Evaluation of the paper substrate and injection volume during µPAD optimization. **A** Amperometric measurements were recorded with µPADs assembled with different paper substrates, such as chromatographic (Chrom), Whatman 40 (W40), Whatman 41 (W41), and Whatman 42 (W42) paper. This experiment was conducted by injecting 2 µL of 50 µmol L^−1^ picric acid in 0.1 mol L^−1^ HCl. **B** Respective graph of *-I*_*p*_ vs paper type. **C** Amperometric signals were obtained with the µPAD by injecting from 1 to 4 µL of 50 µmol L^−1^ picric acid in 0.1 mol L^−1^ HCl. **D** Respective graphs of *-I*_*p*_* vs.* injection volume. Paper substrate: W41. Detection potential: −0.5 V vs Ag/AgCl pseudo-RE

After evaluating the experimental conditions, the thermoplastic chips were combined with a circular paper substrate to generate µPADs (Fig. 1B). Figure S1 shows images of the three-electrode thermoplastic chip and µPAD. The injection (Fig. 1B) is conducted at the center of the µPAD, where the paper-covered WE is positioned. In this configuration, the paper substrate serves exclusively to promote the radial dispersion of the analyte solution after reaching the working electrode surface, enabling new injections. To explain the signal obtained with the µPAD, the amperometric response of a single injection is shown in Figure S2. The current rapidly increases after injection and gradually decreases over time as the analyte solution spreads through the paper. This current decays to the baseline, generating an amperometric response in a peak shape. After background stabilization, new injections are conducted with the µPAD (Fig. 3A).

Figure 3A also shows the influence of the amperometric response of µPADs with different paper substrates. Figure 3B shows that the paper type affects the intensity of the *I*_*p*_, yielding an enhanced response with larger-pore-size filter papers. The pore size is 2.5 µm for W42, 8 µm for W40, and 25 µm for W41. Considering the injection is performed over the paper-covered WE, larger pore sizes increase the analyte solution's accessibility to the electrode's electroactive sites, thereby intensifying the *I*_*p*_ values. Concomitantly, the larger-pore-size paper can provide an enhanced flow rate, rapidly spreading the analyte solution. The literature shows that the flow rate in µPADs can be modulated by changing the paper type, ranging from 0.08 to 0.33 µL s^−1^ [35–38]. The pore size of chromatographic paper is not disclosed by the manufacturer. However, this substrate provided an amperometric response comparable to that of W40. Despite that, the W41 filter paper was selected as the best substrate, given its superior current intensity.

The influence of injection volumes was next evaluated (Fig. 3C). Figure 3D shows that the *I*_*p*_ increases as the injection volume varies from 1 to 2 µL, reaching a maximum. The signal enhancement is associated with the increased number of electroactive species delivered to the electrode surface, thereby maximizing *I*_*p*_ values (Fig. 3D). *I*_*p*_ intensity did not increase for injection volumes greater than 2 µL, probably due to the rapid dispersion of the analyte through the paper substrate after injection. It is important to mention that smaller injection volumes are more susceptible to signal variations (Fig. 3C and 3D) because they are dispensed using a conventional micropipette, making the injection process more difficult to reproduce. Even though ˃2 µL volume slightly enhanced the signal consistency, larger volumes generated broader amperometric peaks (Fig. 3C), decreasing the injection throughput (Fig. 3D). Considering the best compromise between the peak current and injection throughput, 2 µL was selected for subsequent studies.

**Fig. 4:**
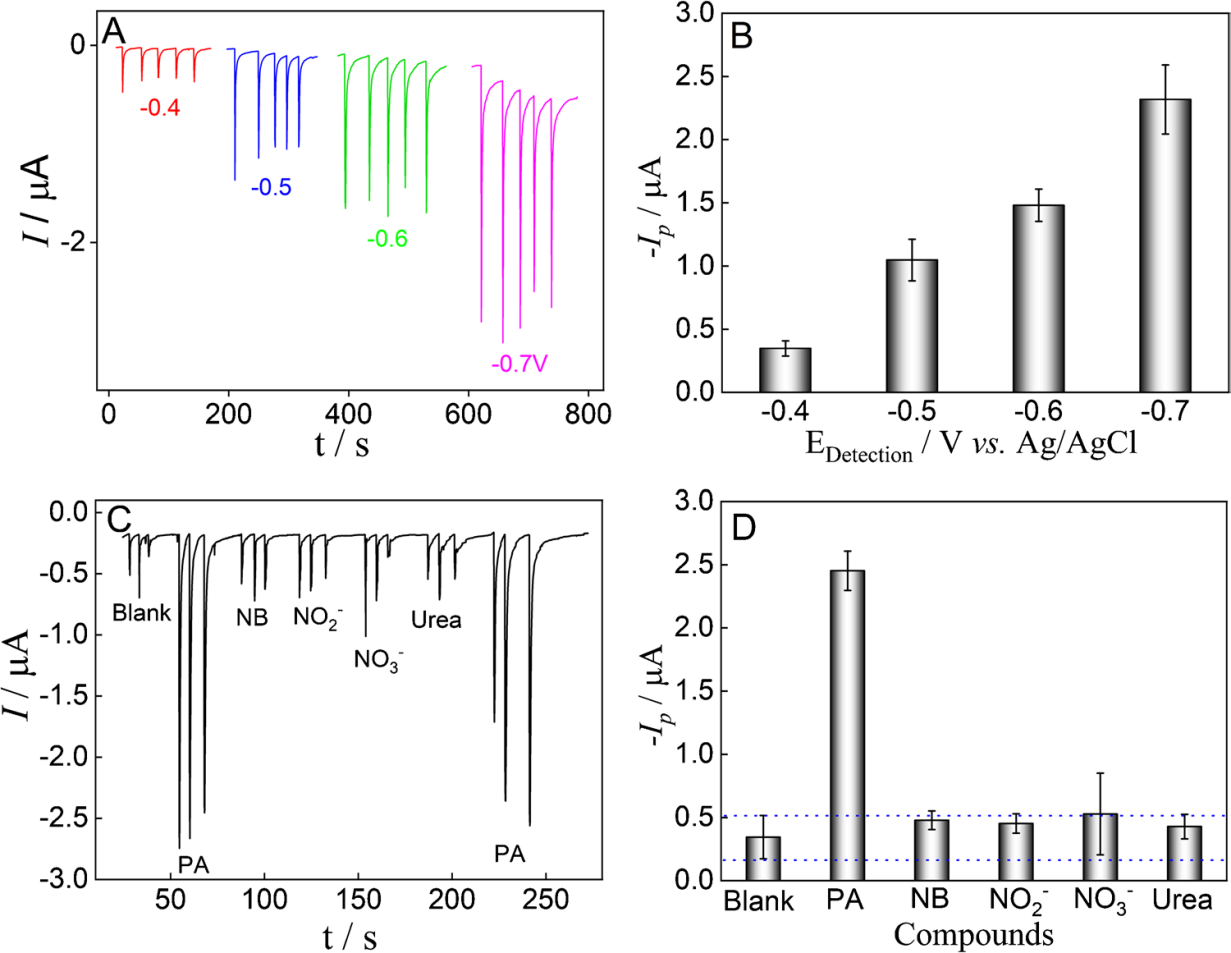
Evaluation of the detection potential and sensor selectivity in the µPAD. **A** Amperometric signals were recorded with a µPAD by evaluating the detection potential from −0.4 to −0.7 V vs Ag/AgCl pseudo-RE. The measurements were obtained by injecting 2 µL of 50 µmol L^−1^ picric acid in 0.1 mol L^−1^ HCl. Paper substrate: W41. **B** Respective graph of *-I*_*p*_ vs detection potential. **C** Amperometric responses were recorded with the µPAD by injecting the blank solution, followed by injections of 50 µmol L^−1^ picric acid and possible interfering compounds at the concentration of 50 µmol L^−1^. PA and NB mean picric acid and nitrobenzene, respectively. Detection potential: −0.5 V vs Ag/AgCl pseudo-RE. Supporting electrolyte: 0.1 mol L^−1^ HCl. **D** Respective graph of *-I*_*p*_ vs possible interfering compounds

The detection potential was next evaluated (Fig. 4A). Increasing the detection potential intensifies the analytical response (Fig. 4B). However, increasing this parameter also increases the background current from 0.03 to 0.41 A, probably due to undesirable parallel reactions, e.g., reduction of dissolved oxygen, occurring under negative-potential conditions. This indicates the susceptibility of co-reducing other species under extremely negative potentials. Considering this behavior, −0.5 V was selected as the detection potential.

It is important to emphasize that this signal enhancement (Figs. 4A and 4B) caused by applying a more negative potential agrees with the results observed by differential pulse voltammetry (Figure S3), which exhibited two reduction peaks at − 0.26 V and − 0.41 V. For amperometric detection in the µPAD, slightly more negative potential (− 0.50 V) was selected to ensure efficient monitoring of both reduction processes. Additionally, this superior potential could also minimize any impact on the electrical resistance introduced by integrating the electrodes with the wet paper structure.

Next, an interfering study (Fig. 4C) was performed by injecting nitrogen compounds at a 1:1 ratio, such as nitrobenzene, nitrite, nitrate, and urea. Figure 4D shows that these species are not reduced on the electrode surface under this potential application, confirming the µPAD selectivity in the presence of these species. Despite exhibiting good selectivity, 2,4,6-trinitrotoluene (TNT), another structurally related nitroaromatic compound, may still interfere with the analytical response of the resulting µPADs because this compound is electrochemically active at the PLA/CB electrode surface [39]. Therefore, knowing the origin of possible sample contamination is essential for correctly interpreting the analytical response and ensuring the selective quantification of picric acid.

### µPAD characterization

**Fig. 5:**
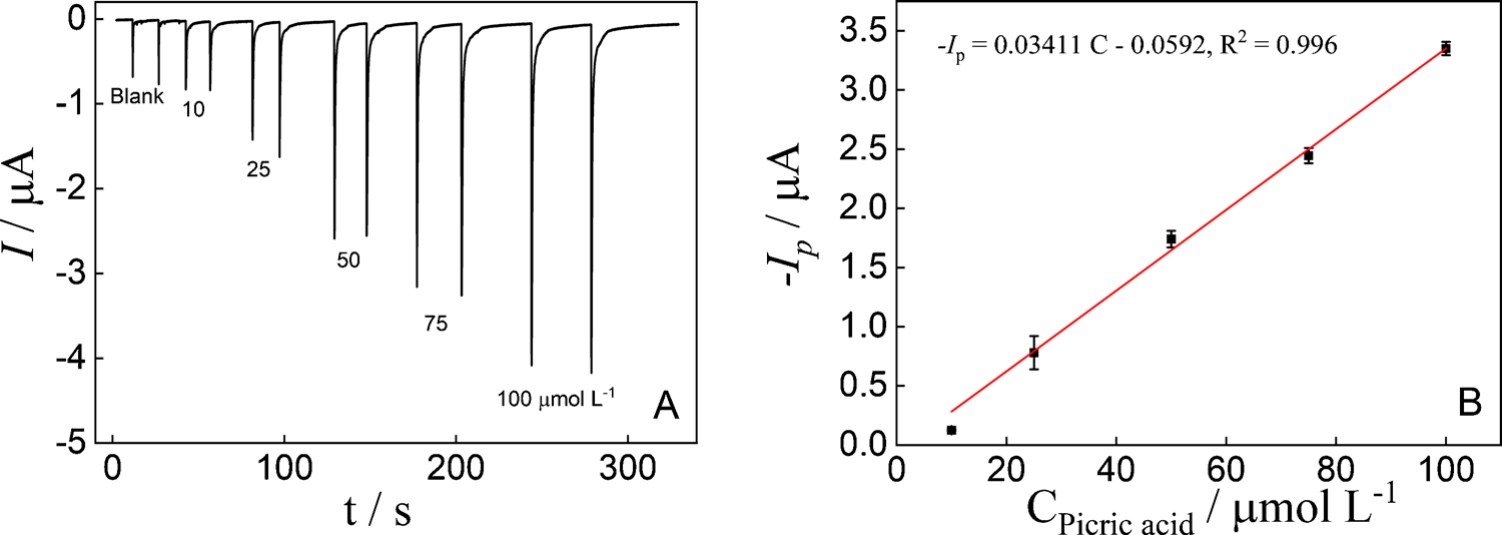
Construction of the analytical curve with the µPAD. **A** Amperometric signals recorded by injecting 2 µL of blank solution and standard solutions of 10, 25, 50, 75, and 100 µmol L^−1^ picric acid. Detection potential: −0.5 V vs Ag/AgCl pseudo-RE. Supporting electrolyte: 0.1 mol L^−1^ HCl. **B** Respective analytical curve. Error bars represent the standard deviation for duplicate measurements

After optimizing the experimental conditions, solutions with different PA concentrations (Fig. 5A) were injected into the µPAD, obtaining a linear response (Fig. 5B) between *I*_*p*_ and PA concentrations from 10 to 100 µmol L⁻^1^, according to the equation -*I*_*p*_ (μA) = 0.03411C—0.0592 (µmol L^−1^), R^2^ = 0.996. Small peaks are observed during blank injections. This behavior is probably associated with perturbations of the electrical double layer at the electrode surface. Before the measurements, the supporting electrolyte solution is injected into the system to establish electrical contact between the electrodes. However, the rapid spreading of this solution through the paper substrate partially carries ionic species away from the working electrode surface, temporarily altering the local ionic environment. When the measurement begins, the applied potential induces the formation of an electrical double layer at the electrode surface with the surrounding electrolyte solution. Under this condition, the blank solution is injected to record its measurement. This injection rapidly increases the ionic concentration at the electrode surface, perturbing this electrical double layer and generating a small current response. Considering the small blank response, the calibration curve was constructed by subtracting the blank current from the current measured for each picric acid standard solution.

The merit figures were calculated from the equations: LOD = 3 Sd (blank)/m and LOQ = 10 Sd (blank)/m. Sd (blank) is the standard deviation of the blank signal (0,056), while m represents the slope of the analytical curve (0.03411). The limit of detection (LOD) and limit of quantification (LOQ) were 5.0 and 16.4 µmol L^−1^, respectively. Considering the limit of quantification was higher than the first point of the calibration curve, 10.0 µmol L^−1^ (Fig. 5) can be a more realistic LOQ value.

The maximum injection number with a single device will depend on the analyte concentration, as a higher concentration will take longer to disperse, resulting in larger peaks (Fig. 5A). To evaluate this parameter, a single device was subjected to consecutive injections (Figure S4A) of 40 µmol L^−1^ picric acid, which is a concentration within the linear range. The µPAD supported thirteen successive injections without signal loss. Additionally, the injections resulted in a relative standard deviation (RSD) of only 5.3%, confirming the injection repeatability. Figure S4B presents the percentage of the peak current as a function of injection number. The peak current remained close to 100% over successive injections, indicating the absence of cross-contamination during sequential measurements. It is important to highlight that no cleaning step was performed to eliminate possible residual analyte solution, confirming its practical utility.

The sample throughput was calculated from the multiple injection study (Figure S4A). The calculation consisted of dividing the peak width (in seconds) by 3600 s (1 h) [30], resulting in (131 ± 14) injections per hour. This estimation represents a good consistency with an RSD of 10,7%. Additionally, this sample throughput is attractive compared to other microfluidic systems [30, 38, 40], taking from 17 to 76 injections per hour. Reproducibility was also evaluated with three different µPADs (Figure S5), demonstrating reliability with an RSD of 9% (*n* = 3).

### Sample analysis

The analytical applicability was then investigated using lake water samples spiked at two concentration levels, such as 50 and 100 µmol L^−1^Although the Occupational Safety and Health Administration (OSHA) has established a Permissible Exposure Limit (PEL) of 0.1 mg m^−3^ for airborne picric acid [41], no specific regulatory threshold has been established for this compound in water. Consequently, the picric acid quantification was motivated by its toxicity instead of meeting regulatory requirements. Considering the absence of picric acid in the respective lake water, this study (Figure S6) was conducted by first injecting the blank and standard solutions, followed by the injection (in triplicate) of the spiked lake water samples. The ability to perform multiple injections into a single device enabled system self-calibration before sample injections, circumventing problems of manual assembly and device-to-device variability [42]. The results are summarized in Table 1. The proposed method provided consistent responses, resulting in recoveries close to 100%. This good recovery percentage confirms the absence of any interference from the lake water matrix. Although the proposed method exhibited limited precision (RSD values ranging from 12.6% to 18.7%), the measurement variability could be reduced by using an electronic micropipette with programmable dispersion [43], as this machine eliminates the analyst dependence during sequential injection, improving the signal consistency. Despite that, a t-test was applied to compare the found concentration values with the added ones. This test suggests the proposed system provides accuracy for PA quantification since t_calculated_ < t_critical_ value at a 95% confidence level. Therefore, the µPAD could serve as a rapid test for PA quantification in environmental samples.

**Table 1:** Recovery obtained with spiked lake water

Added concentration/µmol L^−1^	Found concentration/µmol L^−1^	RSD/%	Recovery/%	t
50	50.2 ± 9.4	18.7	100.4 ± 18.8	0.04
100	100.8 ± 12.7	12.6	100.8 ± 12.7	0.11

t-critical equal to 4.3 with a confidence level of 95% and two degrees of freedom (N—1 = 2). N represents the number of measurements, which is equal to three because of the triplicate injection

After evaluating the analytical applicability of the µPADs, their analytical parameters were also compared with those of other previous systems (Table 2). Our linear range and LOD are within the values reported by other systems, demonstrating that the proposed µPADs offer a competitive analytical response for PA quantification. It is important to emphasize that most reported methods necessitate electrode modification with metallic particles to achieve the required analytical performance, increasing fabrication complexity, cost, and preparation time. In contrast, the proposed method requires no electrode modification or activation step, which simplifies device fabrication and analysis. The main advantages of our devices include ease of integration with paper platforms, offering a practical alternative for microfluidic analyses. Furthermore, the electrochemical systems support multiple sample injections, enhancing the sample throughput. The system integration enables conducting analyses with only 2 μL of sample, reducing chemical use and waste. Unlike other paper-based microfluidic systems [37, 38, 44], our µPAD does not rely on hydrophobic barriers to guide the injected solution, simplifying device fabrication [37, 44]. Moreover, the microfluidic device is enclosed using a PMMA cover and clamps, minimizing contamination during handling. This design also eliminates the need for adhesive tapes to seal the microfluidic system [38], making the assembly process more practical. Therefore, this work offers a straightforward alternative for µPAD fabrication and demonstrates its practical applicability for PA monitoring.

**Table 2:** Merit figures of analytical methods reporting the electrochemical quantification of PA

System	Technique	Linear range/µmol L^−1^	LOD/µmol L^−1^	Reference
Silver/SPE	DPV	200—1100	200	[11]
ZnO/SPE	CV	78—10000	78	[45]
Silver-printed electrodes	LSV	25—250	12	[12]
ZnO/Inkjet-printed paper electrodes	SWV	4—60	4.04	[13]
glove-based sensor	SWV	5—100	0.24	[9]
µPADs	Amperometry	10—100	5.0	This work

*SPE* screen-printed electrodes, *LSV* linear sweep voltammetry, *SWV* square wave voltammetry

## Conclusions

This work reports a straightforward alternative for the quantification of explosive 2,4,6-trinitrophenol (picric acid). The approach involves initially producing a three-electrode thermoplastic chip using 3D printing. This machine eliminates the need for specialized training in 3D-printed device design, affording electrode production at most research laboratories. Additionally, this machine could also assist professors in electrochemistry didactic studies. After characterizing the resulting chips, they were easily integrated with a microfluidic paper-based analytical device (µPAD), enabling sequential injections into a single system. In addition to enabling multiple injections, the system requires only 2 µL of sample for analysis. µPAD fabrication also showed practicality, dispensing the need for cleanroom facilities typically required for microfluidic system development. The resulting device exhibited good selectivity and enabled the self-calibration before sample analyses, eliminating problems normally observed with manual device assembly. Moreover, the analytical system provided accurate PA quantification, as confirmed by recovery studies. Therefore, this work presents an attractive and practical analytical platform for monitoring picric acid and opens possibilities for other exciting applications.

## Supplementary Information

Below is the link to the electronic supplementary material.

Supplementary file1 (DOCX 623 KB)

## Data Availability

The authors declare that the data supporting the findings of this study are available within the paper.
