# Supplementary material for: Paper-based microfluidic devices for electrochemical quantification of the explosive picric acid
Source: Mikrochim Acta. 2026 Jul 31;193(8):585. doi: 10.1007/s00604-026-08311-0 (PMC13427850; doi:10.1007/s00604-026-08311-0)
Supplement: Supplementary file 1 — Supplementary file1 (DOCX 623 KB) [file 604_2026_8311_MOESM1_ESM.docx]

Supplementary Material

**Paper-Based Microfluidic Devices for Electrochemical Quantification of the Explosive Picric Acid**

Julia de Oliveira Cardoso^a^, Lauro Antonio Pradela Filho^a,b*^, and Thiago Regis Longo Cesar da Paixão^a*^

*^a^Institute of Chemistry, Department of Fundamental Chemistry, University of São Paulo, 05508-000, São Paulo-SP, Brazil;*

*^b^Institute of Chemistry, Federal University of Mato Grosso do Sul, 79074-460, Campo Grande - MS, Brazil*

*Corresponding Author

E-mail Address: [lauro_pradela@ufms.br](mailto:lauro_pradela@ufms.br), [trlcp@iq.usp.br](mailto:trlcp@iq.usp.br)

Phone: +55 11 30919150

**
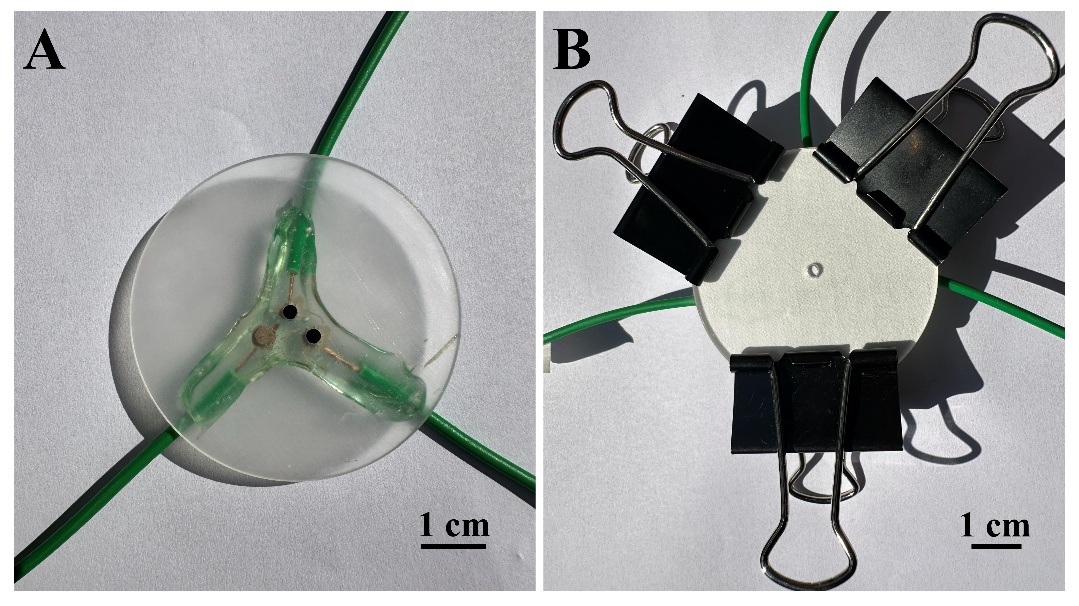
**

**Figure S1.**  Images of the A) three-electrode thermoplastic chip and B) microfluidic paper-based analytical devices (µPAD).

**Figure S2.** Evaluation of the analytical signal generated during the injection of 2 µL of 50 µmol L^-1^ picric acid in 0.1 mol L^-1^ HCl. The amperometric measurement was recorded with µPADs assembled with W40. Detection potential: -0.5 V vs Ag/AgCl pseudo-RE.

**Figure S3.** Differential pulse voltammograms were recorded in HCl 0.1 mol L^-1^ in the absence and presence of 50 µmol L^-1^ picric acid using Ag/AgCl pseudo-RE. The blue dashed line shows the detection potential selected for the µPAD from the amperometric results (Figures 4A and 4B).

**Figure S4.** Evaluation of the signal under successive injections. A) Amperometric signals were recorded by injecting 2 µL of 40 µmol L^-1^ picric acid in 0.1 mol L^-1^ HCl. Paper substrate: W41. Detection potential: -0.5 V vs Ag/AgCl pseudo-RE. B) Graphic of normalized peak current *vs.* injection number. The normalization was performed with the peak current of the first injection considered as 100 %.

**Figure S5.**  A reproducibility study was conducted with three different µPADs. Amperometric signals were recorded at -0.5 V by injecting 2 µL of 50 µmol L^-1^ picric acid in 0.1 mol L^-1^ HCl. Paper substrate: W41. Detection potential: -0.5 V vs Ag/AgCl pseudo-RE.

**Figure S6.** A) Amperometric signals for the PA standard solution, followed by injections of spiked lake water samples. Injection volume: 2 µL. Supporting electrolyte: 0.1 mol L^-1^ HCl. Paper substrate: W41. Detection potential: -0.5 V vs Ag/AgCl pseudo-RE. B) Respective analytical curve.
